# Professional Continuous Glucose Monitoring for the Identification of Type 1 Diabetes Mellitus Among Subjects With Insulin Therapy

**DOI:** 10.1097/MD.0000000000000421

**Published:** 2015-01-26

**Authors:** Yin-Chun Chen, Yu-Yao Huang, Hung-Yuan Li, Shih-Wei Liu, Sheng-Hwu Hsieh, Chia-Hung Lin

**Affiliations:** From the Division of Endocrinology and Metabolism (Y-CC, Y-YH, S-HH, C-HL), Department of Internal Medicine, Chang Gung Memorial Hospital; Division of Endocrinology and Metabolism (H-YL), Department of Internal Medicine, National Taiwan University Hospital; Department of Chemical Engineering (S-WL), National Taiwan University, Taipei; Graduate Institute of Clinical Medical Sciences (C-HL), Chang Gung University, Taoyuan, Taiwan.

## Abstract

The identification of type 1 diabetes in diabetic subjects receiving insulin therapy is sometimes difficult. The purpose of this study is to evaluate whether results of professional continuous glucose monitoring can improve the identification of type 1 diabetes.

From 2007 to 2012, 119 adults receiving at least twice-daily insulin therapy and professional continuous glucose monitoring were recruited. Type 1 diabetes was diagnosed by endocrinologists according to American Diabetes Association standards, including a very low C-peptide level (<0.35 pg/mL) or the presence of diabetic ketoacidosis. Continuous glucose monitoring was applied for 3 days.

Among 119 subjects, 86 were diagnosed with type 1 diabetes. Subjects with type 1 diabetes were younger (33.8 vs 52.3 years old, *P* < 0.001), had lower body mass index (BMI, 21.95 vs 24.42, *P* = 0.003), lower serum creatinine (61.77 vs 84.65 μmol/L, *P* = 0.001), and higher estimated glomerular filtration rate (108.71 vs 76.48 mg/mL/min/1.73m2, *P* < 0.001) than subjects with type 2 diabetes. Predictive scores for identification of type 1 diabetes were constructed, including age, BMI, average mean amplitude of glucose excursion in days 2 and 3, and the area under the curve of nocturnal hyperglycemic and hypoglycemic states. The area under the receiver operating characteristic curve was 0.90. With the cutoff of 0.58, the sensitivity was 86.7% and the specificity was 80.8%. The good performance was validated by the leave-one-out method (sensitivity 83.3%, specificity 73.1%).

Professional continuous glucose monitoring is a useful tool that improves identification of type 1 diabetes among diabetic patients receiving insulin therapy.

## INTRODUCTION

It is sometimes difficult to distinguish between type 1 diabetes and type 2 diabetes in patients with intensive insulin therapy. Patients with type 1 diabetes have an absolute requirement for insulin therapy. However, many patients with type 2 diabetes lose β-cell function over time and require insulin for glucose control. Differentiation is especially challenging in subjects receiving insulin therapy, and current criteria and performance of diagnostic tests are not perfect. One of the most characteristic features in subjects with type 1 diabetes is having greater glucose variability than subjects with type 2 diabetes. Higher postprandial glucose excursion and frequent hypoglycemia is often an obvious pattern in patients with type 1 diabetes.^1^

Continuous glucose monitoring (CGM) systems are devices that use an electrochemical enzymatic sensor to measure the glucose content in interstitial fluid at regular intervals, and results correlate well with plasma glucose measurements. Interstitial fluid is accessed by a needle sensor inserted subcutaneously.^2^ In “professional” CGM, the device is owned and controlled by medical professionals and patients receive no information while wearing the device. Since there are either minimal behavior changes or none at all in response to the results of professional CGM, this method is more likely to observe the patients’ actual glucose excursions. Results can be determined in a clinician's office and graphed, providing useful information, including the extent of within-day and between-day variations in blood glucose, and the frequency of unrecognized hypoglycemia.

To the best of our knowledge, this is the first study to evaluate whether CGM measurement of glucose excursions can improve the identification of type 1 diabetes. If the pattern of glucose excursion is favored of type 1 diabetes mellitus (DM), the titration of insulin dose in these patients should be more careful than in those with type 2 DM to avoid hypoglycemia. Therefore, this study was designed to evaluate data from professional CGM in subjects receiving insulin therapy and determine if this monitoring method can improve the identification of type 1 diabetes.

## MATERIALS AND METHODS

### Subjects

A total of 119 diabetic patients receiving professional CGM were enrolled for the study between 2007 and 2012. All included patients met the following criteria: diagnosis of type 1 or type 2 diabetes for more than 1 year and at least twice-daily insulin therapy, including premixed insulin or basal–bolus regimen. The exclusion criteria were a recent history of steroid or alcohol abuse; serious cardiovascular disorders; ongoing influenza, autoimmune disease, or other metabolic disorders; and pregnant or lactating women. Type 1 diabetes was diagnosed by endocrinologists according to the American Diabetes Association standards, including a very low C-peptide level (<0.35 pg/mL) or presence of diabetic ketoacidosis (DKA).^3^ The diagnosis of type 1 diabetes in the patients was confirmed by the peer-reviewed process and the type 1 diabetes patients were all registered in the National Health Insurance database in Taiwan. Informed consent was obtained from each subject. The study was approved by the institutional review board of Chang Gung Memorial Hospital.

### Continuous Glucose Monitoring

The MiniMed Solutions: CGMS sensor (MMT-7310, version 3.0B (3.0.116); Medtronic, Minneapolis, MN) was used to retrieve CGM data. Glucose variability was evaluated by the standard deviation of plasma glucose^4^ and the mean amplitude of glucose excursion (MAGE)^5^ of all glucose values obtained within 3 days. The areas under the curve (AUCs) of a glucose level above 10 mmol/L (AUC_high_) and below 3.89 mmol/L (AUC_low_) were calculated as measures of hyperglycemic and hypoglycemic states, respectively. A hypoglycemic event was defined as a glucose concentration below 3.89 mmol/L. Information recorded between 0000 and 0600 hours was defined as nocturnal occurrence.

### Statistical Analysis

Descriptive data are presented as means and standard deviations for continuous variables, and as percentages for nominal variables. Student *t* tests and χ^2^ tests were used to identify differences between subjects with type 1 and type 2 diabetes. Odds ratios and 95% confidence intervals for type 1 diabetes were derived from logistic regression models using subjects with type 2 diabetes as the reference group (odds ratio = 1). Three predictive scores were constructed based on the multiple logistic regression models, using the regression coefficients as the weight for the dependent variables. Age, body mass index (BMI), the average of MAGE on days 2 and 3, and the AUC of nocturnal hyperglycemic and hypoglycemic states were included in different predictive scores. The diagnostic performance was evaluated by the area under the receiver operating characteristic (ROC) curve. The optimal cutoff point was derived from the ROC curve with shortest distance to sensitivity = 1 and 1 − specificity = 0. The sensitivity is the probability that the prediction will be positive for subjects with type 1 diabetes. The specificity is the probability that the prediction will be negative for subjects without type 1 diabetes. A *P* value less than 0.05 was considered to indicate statistical significance. All statistical analyses were performed using Stata/SE 9.0 for Windows (Stata Corp, College Station, TX).

## RESULTS

A total of 119 diabetic patients (43 men and 76 women aged 10–81 years) were enrolled and 86 were diagnosed with type 1 diabetes. Patients’ clinical variables are summarized in Table 1. Subjects with type 1 diabetes were younger (33.8 vs 52.3 years old, *P* < 0.001), had lower BMI (21.95 vs 24.42, *P* = 0.003), lower serum creatinine (61.77 vs 84.65 μmol/L, *P* = 0.001), and higher estimated glomerular filtration rate (108.71 vs 76.48 mg/[mL/min/1.73m2], *P* < 0.001) than subjects with type 2 diabetes.

**TABLE 1:**
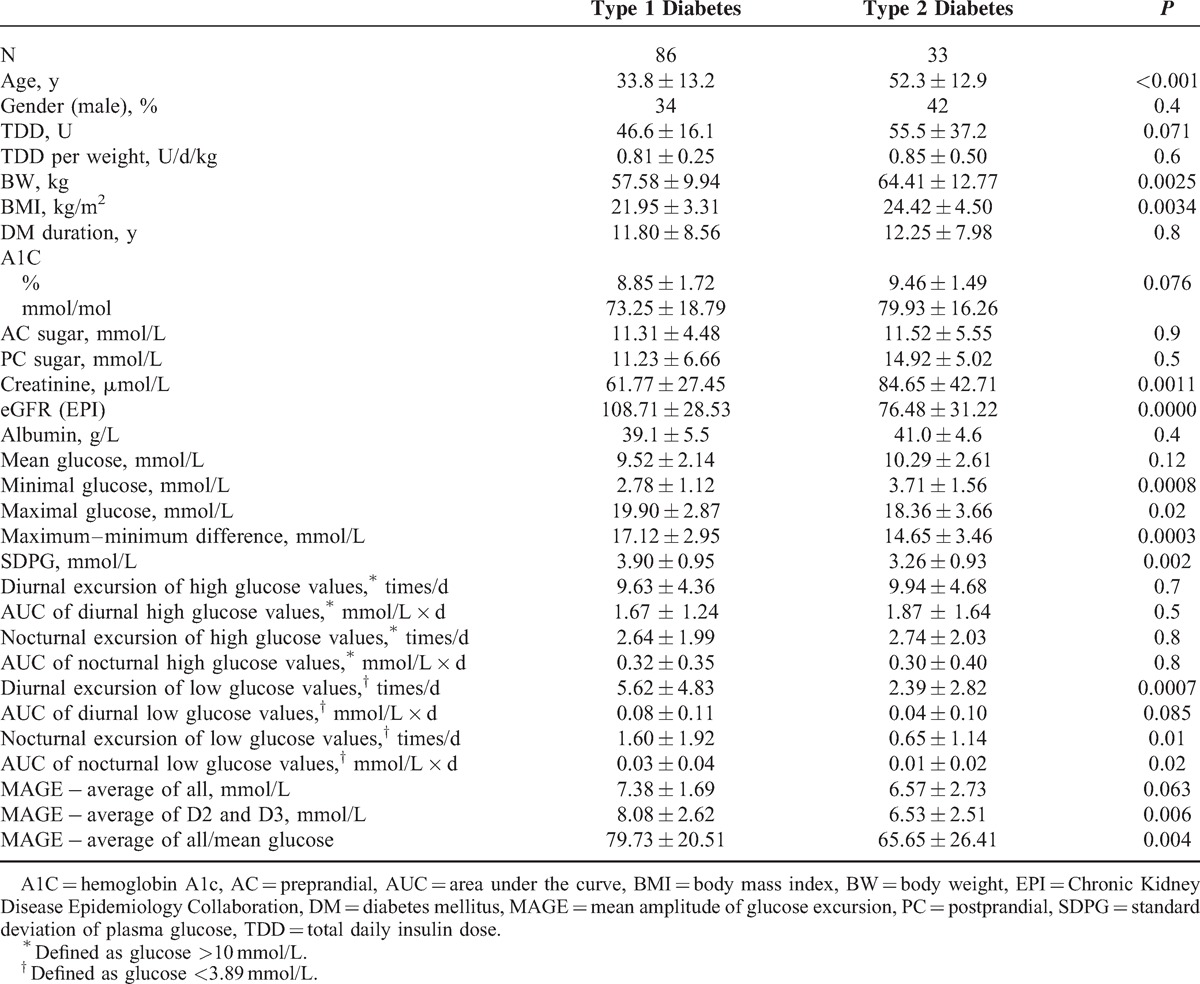
Clinical Characteristics and Features of Subjects With Type 1 and Type 2 Diabetes Receiving Professional Continuous Glucose Monitoring

**FIGURE 1:**
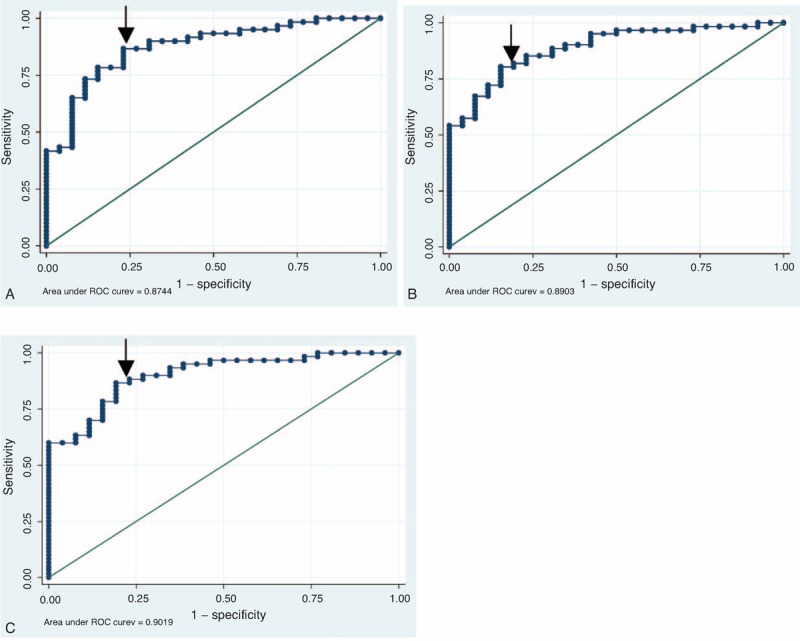
The ROC curve of (A) score 1, (B) score 2, and (C) score 3. Arrow indicates the optimal cutoff point. ROC = receiver operator characteristic.

In Table 2, higher average MAGE, higher AUC of nocturnal high glucose, and higher AUC of nocturnal low glucose were associated with greater chance of having type 1 diabetes after adjusting for age and BMI. In Table 3 and Figure 1, these 3 predictive scores demonstrated good performance for identifying type 1 diabetes (area under the ROC curve, 0.874–0.902). Incorporation of the average MAGE (D2 and D3) and AUC of nocturnal high/low glucose improved the area under the ROC curve from 0.848 (age and BMI only) to 0.902. Using optimal cutoff values, sensitivity ranged from 80.3% to 86.7% and specificity ranged from 76.9% to 84.6%. Similar results were validated by the leave-one-out cross-validation method, with sensitivity 78.7% to 86.7% and specificity 73.1% to 76.9%.

**Table 2:**
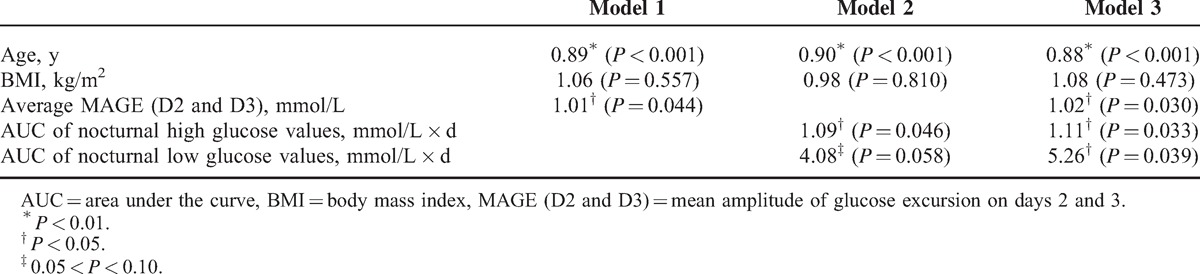
Odds Ratios (*P* Values) of Clinical Characteristics and Features of Subjects on Continuous Glucose Monitoring for Type 1 Diabetes (vs Type 2 Diabetes) by Multiple Logistic Regression Models

**Table 3:**
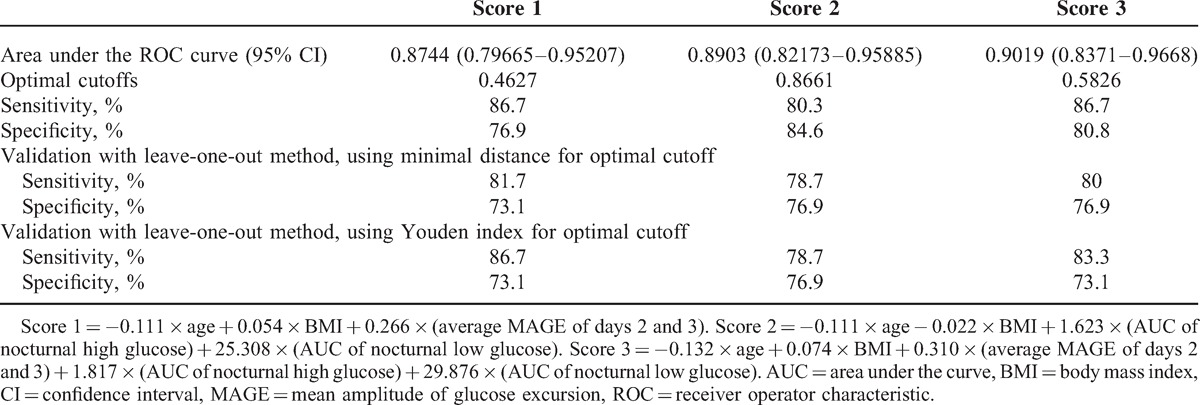
The Performance of Predictive Scores to Differentiate Subjects With Type 1 Diabetes From Subjects With Type 2 Diabetes

## DISCUSSION

This is the first study to evaluate the use of professional CGM for the identification of type 1 diabetes in diabetic subjects receiving insulin therapy. We found that MAGE, nocturnal high glucose, and nocturnal low glucose were associated with type 1 diabetes. Three predictive scores were constructed, including age, BMI, MAGE, and nocturnal high and low glucose values. These predictive scores performed well in identifying type 1 diabetes, suggesting that professional CGM is useful for identifying type 1 diabetes in insulin users.

This study applied an observational design to evaluate the performance of professional CGM for diabetic patients receiving insulin in Chang Gung Memorial Hospital. Professional CGM was administered for poor glucose control. Even though all patients used advanced insulin therapy with either premixed or basal–bolus insulin regimens, the clinicians still needed professional CGM for clinical adjustments. Comparisons between type 1 and type 2 diabetic patients elucidated obvious patterns with higher glucose excursion and more frequent hypoglycemic states. Our data demonstrated that the use of data derived from professional CGM provided a predictive advantage for type 1 diabetes among diabetic patients with advanced insulin therapies. Although the characteristics of younger age, lower BMI, lower creatinine levels, and higher eGFRs were easily detected in clinical practice, the 3 risk scores of the prediction model demonstrated good performance.

Data from the present study are the first to distinguish between type 1 and type 2 diabetes based on performing professional CGM. No other set of criteria or diagnostic tests can consistently distinguish between type 1 and type 2 diabetes. Type 1 diabetes is suggested by the presence of circulating, islet-specific, pancreatic autoantibodies against glutamic acid decarboxylase (GAD65), the 40K fragment of tyrosine phosphatase (IA2), insulin, and/or zinc transporter 8 (ZnT8). However, the absence of pancreatic autoantibodies does not rule out the possibility of type 1 DM. The use of professional CGM in insulin users is able to reinforce the control of blood glucose levels. Using the scoring system can guide clinicians in insulin treatment decisions. Among patients who are likely to have type 1 diabetes, more careful insulin dose titration is necessary due to the high risk of hypoglycemia. Based on the risk scores determined in the present study, we have identified an additional effective tool for clinical practice to differentiate patients with vague diagnoses.

Type 1 diabetes is characterized primarily by insulin deficiency, whereas type 2 diabetes is characterized primarily by insulin resistance with relative insulin deficiency.^6–11^ The lack of insulin reservoir in type 1 diabetes often results in greater glycemic excursion, which may present as extremely high and low glucose levels.^1^ Indeed, we found that subjects with type 1 diabetes had greater average MAGE, higher AUC of nocturnal high glucose, and higher AUC of nocturnal low glucose. Our findings indicate that these data improved the identification of type 1 diabetes in insulin users. The nocturnal period is the most undisturbed time for glycemia, since there is no food intake and only a few people exercise during the night. The AUC also accounts for the duration of low or high glucose, which could magnify the difference between type 1 and type 2 diabetes. Additionally, these nocturnal patterns are not totally recorded if patients use glucometers,^12^ which is in contrast to the nocturnal pattern identification of professional CGM.

The limitation of this study is the lack of correlations between professional CGM data and C-peptide level, as well as with presence of immunological markers of β-cell destruction. Indeed, low C-peptide level could explain the greater glucose level variability in CGM. In the diagnosis of type 1 diabetes, an undetected C-peptide level (<0.35 pg/mL) (fasting and after stimulation) was essential in this study. The anti-GAD65 and IA-2 antibody titers also diminish over time and are often negative in Asians.^13,14^ Therefore, the measurement of C-peptide level and autoantibodies in patients with type 1 DM was often undetectable. Otherwise, the measurement of C-peptide level and antibodies in patients with type 2 diabetes was not routinely done in our clinical practice. Clinically, family history of type 2 diabetes, high BMI, and other signs of insulin resistance without evidence of DKA could make the diagnosis of type 2 diabetes precisely. In the long-standing type 2 diabetes, C-peptide level is also decreasing over time and these patients need insulin therapy but the variations of their glucose level are less than type 1 diabetes due to the insulin resistance. There is limited study to explore this difference. It is highly related to the risk of hypoglycemia while titrating insulin dose in these patients with advanced insulin therapy.

## CONCLUSIONS

We found that professional CGM is useful for the identification of type 1 diabetes among diabetic subjects receiving insulin therapy. MAGE and AUC of nocturnal hyperglycemic and hypoglycemic states determined by CGM can improve the diagnostic performance beyond traditional clinical factors.
